# Thermal conductivity in porous silicon nanowire arrays

**DOI:** 10.1186/1556-276X-7-554

**Published:** 2012-10-06

**Authors:** Jeffrey M Weisse, Amy M Marconnet, Dong Rip Kim, Pratap M Rao, Matthew A Panzer, Kenneth E Goodson, Xiaolin Zheng

**Affiliations:** 1 Department of Mechanical Engineering, Stanford University, Stanford, CA, 94305, USA; 2 KLA-Tencor Corporation, Milpitas, CA, 95035, USA

**Keywords:** Thermal conductivity, Silicon nanowires, Porous silicon, Thermoreflectance

## Abstract

The nanoscale features in silicon nanowires (SiNWs) can suppress phonon propagation and strongly reduce their thermal conductivities compared to the bulk value. This work measures the thermal conductivity along the axial direction of SiNW arrays with varying nanowire diameters, doping concentrations, surface roughness, and internal porosities using nanosecond transient thermoreflectance. For SiNWs with diameters larger than the phonon mean free path, porosity substantially reduces the thermal conductivity, yielding thermal conductivities as low as 1 W/m/K in highly porous SiNWs. However, when the SiNW diameter is below the phonon mean free path, both the internal porosity and the diameter significantly contribute to phonon scattering and lead to reduced thermal conductivity of the SiNWs.

## Background

Silicon with a high density of nanoscale features such as interfaces, porosity, and impurities can have thermal conductivities (*κ*) up to three orders of magnitude lower than that of bulk Si through enhanced phonon scattering [1-17]. For example, the thermal conductivity of nanoporous bulk Si generally decreases with increasing porosity and decreasing pore size [1-9] and, with high porosity, approaches the amorphous limit (0.2 to 0.5 W/m/K) [1-3]. Similarly, silicon nanowires (SiNWs) with diameters significantly smaller than the bulk phonon mean free path (*Λ* ≈ 100 to 300 nm at 300 K) were reported to have thermal conductivity values as low as 0.76 W/m/K due to strong phonon scattering at the SiNW boundary [10,11]. Introducing surface roughness to the SiNWs leads to additional phonon scattering at length scales even smaller than the NW diameter [12-16]. However, there have been few investigations on the combined effects of external dimensions and internal porosity on the thermal conductivity values of SiNWs. In this work, we report the effects of internal porosity on the thermal conductivity of SiNWs of two different diameters that allow the phonon propagation to span the range from ballistic to diffusive thermal transport (*d*_avg_ ≈ 350 and 130 nm) by measuring the thermal conductivity of vertically aligned SiNW arrays using nanosecond transient thermoreflectance (TTR). As opposed to measurements of individual SiNWs, measurements of arrays of SiNWs offer the advantage of averaging out the inherent thermal conductivity variations that are caused by differences in SiNW diameter, surface roughness, and defects within the arrays.

## Methods

**Figure 1:**
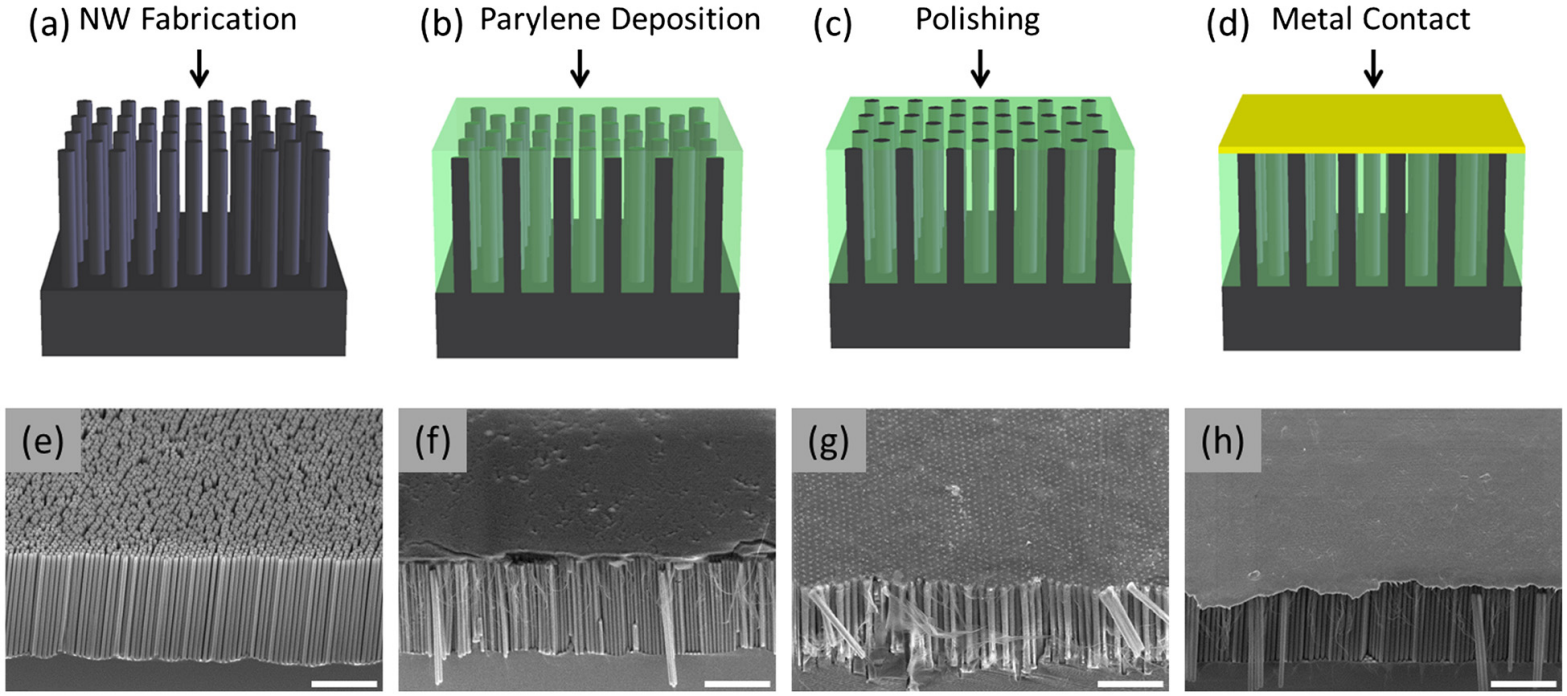
**Fabrication of the vertically aligned SiNW arrays for the nanosecond thermoreflectance measurements.** (**a**,**e**) SiNW arrays are formed using the top-down etching. (**b**,**f**) Parylene is conformally deposited in between NWs and acts as a mechanical scaffold for the top metal transducer layer. (**c**,**g**) The SiNW tips are exposed by chemical mechanical polishing to ensure good thermal contact between the SiNWs and the metal film, and (**d**,**h**) a metal film is deposited over the SiNW array. The scale bars on the SEM images are 5 μm.

The vertically aligned SiNW arrays are fabricated using a four-step preparation process illustrated in Figure 1. Two sets of vertically aligned SiNW arrays with different diameters are fabricated (Figure 1a,e) using top-down etching techniques to achieve a range of porosities (Table 1). For the first set, the diameter (*d*_avg_*≈* 300 to 350 nm) and density of the SiNWs are controlled by nanosphere lithography [18]. Specifically, a monolayer of SiO_2_ spheres is deposited using the Langmuir-Blodgett method onto Si wafers (p-type with boron dopant atoms, (100)) and used as a mask for the subsequent etching steps. The internal porosity of the SiNWs is varied from nonporous to highly porous by changing the etching methods and conditions [19-21]. Nonporous SiNWs are formed by deep reactive ion etching (DRIE), and the resulting SiNWs have slightly smaller diameters (*d*_avg_ ≈ 300 nm) than the spheres used as the etch mask [22]. Porous SiNW arrays are fabricated by metal-assisted chemical etching (MACE) in a solution of 4.8 M HF and 0.3 M H_2_O_2_, and the porosity is controlled by varying the metal catalyst and wafer doping concentrations [19-21,23-25]. For low-porosity nanowires, the catalyst layer consists of a 15-nm Ag film covered by 5-nm Au, while for the moderate to highly porous nanowires, a 50-nm Ag film is used as the catalyst and the initial wafer doping concentration is varied. The second set of SiNWs, with generally smaller diameters, is fabricated using a two-step MACE process with silver salts [19,20,23,26,27]. First, the Ag film is deposited using a solution of 0.005 M AgNO_3_ and 4.8 M HF for 1 min. Then, the SiNWs are formed by etching in a solution of 4.8 M HF with various concentrations of H_2_O_2_ (0.15, 0.30, 0.60, and 1.20 M) to adjust the SiNW porosity [19,20,23,26,27]. The resulting SiNWs have an average diameter of 130 nm, but there is significant diameter variation within the SiNW array (*d ≈* 20 to 300 nm). For all the samples, the SiNW length is approximately 10 μm.

**Table 1:** Summary of SiNW arrays with varied diameters and porosities

	**Diameter control**	**Porosity control**
**Set 1**	**Nanosphere lithography**	**Etching method and doping concentration**
	*d*_avg_*≈* 300 to 350 nm	Nonporous: DRIE
	VF_DRIE_ = 21% to 23%	Low porosity: Ag/Au MACE
	VF_MACE_ = 45% to 60%	Moderate porosity: Ag MACE, lightly doped
		High porosity: Ag MACE, heavily doped
**Set 2**	**Silver salts**	**MACE etchant solution**
	*d*_avg_*≈* 130 nm	Low porosity, 0.15 M H_2_O_2_
	VF = 26% to 35%	High porosity, 1.2 M H_2_O_2_

Following the formation of the SiNW arrays, the gaps between SiNWs are completely filled with parylene N (poly-para-xylylene; Figure 1b,f), which has a thermal conductivity significantly lower than the SiNWs (*K*_parylene_ = 0.125 W/m/K) and a high melting temperature (*T*_m_ ≈ 410°C). The parylene filling quality is inspected by examining multiple freshly cut cross sections under a scanning electron microscope (SEM), and no parylene voids are observed. The SiNW tips are subsequently exposed via chemical mechanical polishing to remove the parylene covering the SiNWs (Figure 1c,g) that facilitates the SiNWs to form a good thermal contact with the top metal film. Finally, a 15-nm Cr layer (for adhesion) and a 500-nm Cu layer are deposited by electron beam evaporation on top of the SiNW tips to form a flat, reflective transducer layer for the thermoreflectance measurements (Figure 1d,h).

The thermal conductivity of the vertical SiNW arrays is measured at room temperature by nanosecond TTR; the details of which can be found in Panzer et al. [28]. Briefly, the metal transducer layer that is deposited on the parylene-filled SiNW array is heated by a 3-mm diameter, 532-nm wavelength, 6-ns pulse from a Nd:YAG laser at a frequency of 10 Hz. The reflected intensity of the probe laser (*d* ≈ 20 μm, 10 mW, 658 nm, continuous wave) is directly correlated to the temperature of the metal layer that is affected by the thermal conductivity of the SiNW/parylene composite. The thermal conductivity of the SiNW/parylene composite and its interface thermal resistance at the top metal layer are extracted using a two-parameter fit of the measured temperature decay trace (normalized by the maximum temperature) to the solution of a one-dimensional heat diffusion equation for a multilayer stack with surface heating. The volumetric heat capacity of the film (*C*_v,composite_) is assumed to be the volumetric average of the heat capacity of parylene (*C*_v,parylene_) and bulk silicon (*C*_v,Si_): *C*_v,composite_ = VF · *C*_v,Si_ + (1 − VF) · *C*_v,parylene_, where VF is the volume fraction of SiNWs within the composite. The VF of SiNWs within each array is measured directly from top-view SEM images of the film by setting a brightness threshold to define the edge of SiNWs. The average thermal conductivity of an individual SiNW within the array is calculated from the extracted film thermal conductivity (*K*_composite_) using an effective medium model: *K*_NW_ = *K*_composite_ − (1 − VF)*K*_parylene_/*VF*, where *K*_NW_ and *K*_parylene_ are the thermal conductivities of the SiNWs and parylene, respectively. In this model, SiNW arrays are treated as thermal resistors in parallel with the parylene matrix. The uncertainty of the extracted *k*_NW_ is calculated through an error propagation analysis given by the following equation:

(1) $$\Delta k_{\mathrm{NW}}=\sqrt{{\left(\frac{\partial k_{\mathrm{NW}}}{\partial k_{\mathrm{film}}}\Delta k_{\mathrm{film}}\right)}^{2}+{\left(\frac{\partial k_{\mathrm{NW}}}{\partial VF}\Delta VF\right)}^{2}+{\left(\frac{\partial k_{\mathrm{NW}}}{\partial k_{\mathrm{parlyene}}}\Delta k_{\mathrm{parlyene}}\right)}^{2}}$$

where Δ*k*_parylene_ is the thermal conductivity variation from the literature. Δ*k*_film_ and ΔVF are the measured spot-spot variation in the same type of samples. Detailed error analysis data for all the data reported here can be found in Additional file 1.

## Results and discussion

**Figure 2:**
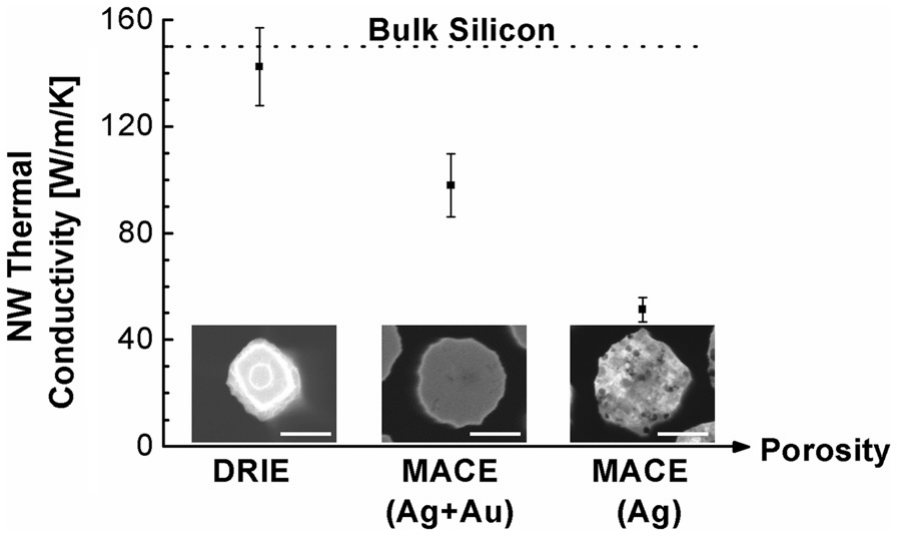
**Thermal conductivity of large-diameter SiNWs (approximately 350 nm; 10**^**14**^**cm**^**−3**^**p-type doping).** The thermal conductivity with three levels of porosity, corresponding to different etching conditions, is shown. The thermal conductivity decreases significantly with increasing porosity. The inset images show the top view of the SiNWs, and the scale bars are 200 nm.

The thermal conductivity for the SiNWs with large diameters (*d*_avg_ ≈ 300 to 350 nm) demonstrates a clear decrease with increasing porosity (Figure 2). The thermal conductivity of nonporous SiNWs, though with rough surfaces, is 142 ± 13 W/m/K, which is very close to that of bulk Si (*κ* ≈ 150 W/m/K). This suggests that for large-diameter SiNWs, surface roughness at this depth and periodicity does not cause effective phonon-external boundary scattering and therefore has little effect on the thermal conductivity. On the other hand, the internal porosity of SiNWs significantly reduces the thermal conductivity from 142 W/m/K for the nonporous SiNWs to 98 W/m/K (Au/Ag-MACE) and 51 W/m/K (Ag-MACE) for the increasingly porous SiNWs.

**Figure 3:**
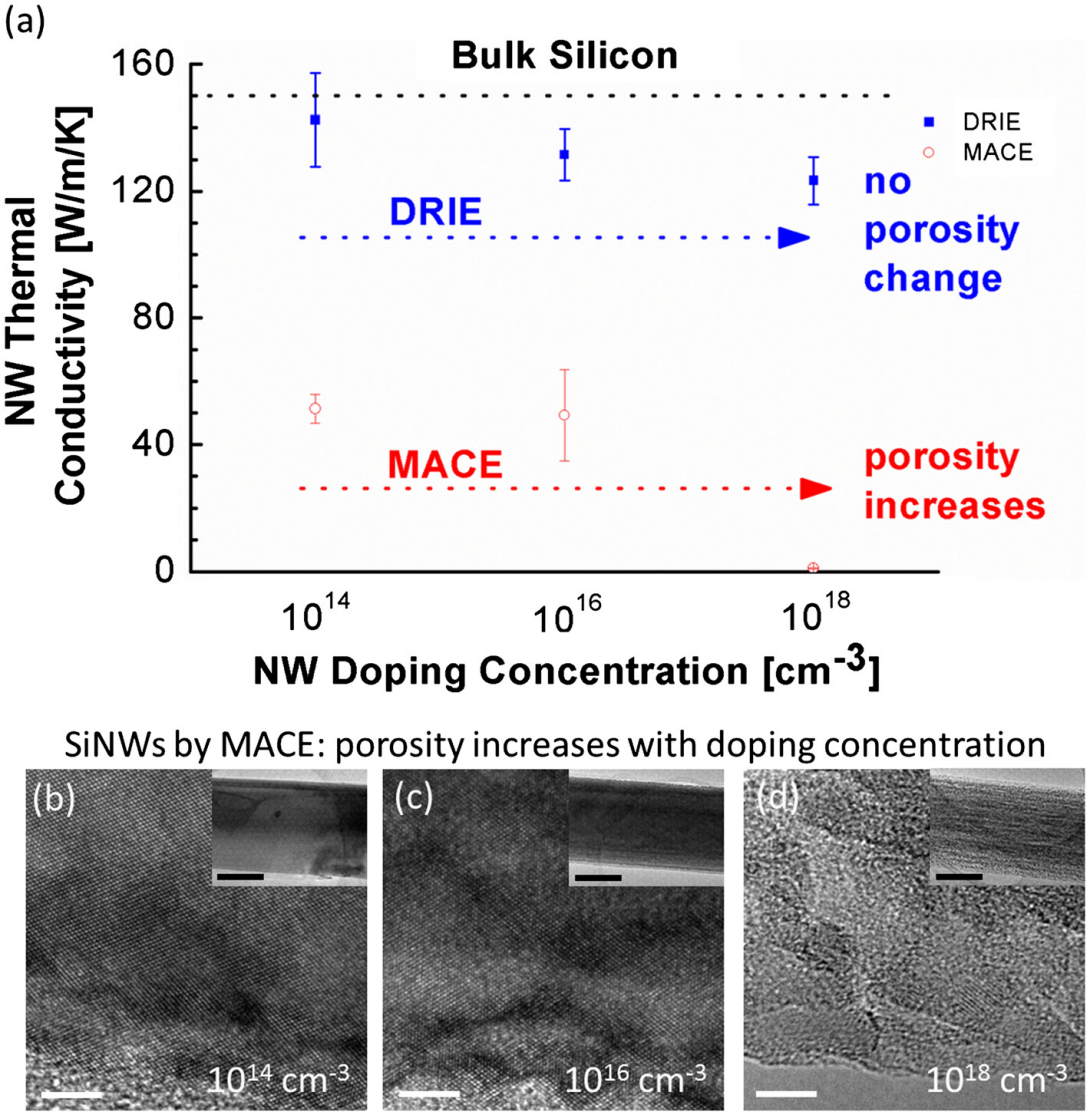
**Thermal conductivity of large-diameter nonporous and porous SiNW arrays.** (**a**) Thermal conductivity of nonporous and porous SiNW arrays of large diameters as a function of doping concentrations. TEM images show the relative porosity for Ag-MACE SiNW arrays fabricated with doping concentrations of (**b**) 10^14^, (**c**) 10^16^, and (**d**) 10^18^ cm^−3^. The scale bars on the TEM and inset TEM images are 5 and 200 nm, respectively. The uncertainty bar for the MACE nanowires with a doping concentration of 10^18^ cm^−3^ is on the order of the data point marker size.

The thermal conductivity of large-diameter SiNW arrays (*d*_avg_ ≈ 350 nm) with three different p-type boron dopant atom concentrations (10^14^, 10^16^, and 10^18^ cm^−3^) is further investigated for both nonporous and porous NWs (Figure 3). The thermal conductivity of nonporous SiNWs decreases slightly with increasing doping concentration due to the increased phonon-impurity scattering, similar to bulk Si [29,30]. Conversely, the thermal conductivity of porous SiNWs drops to about 1 W/m/K when the doping concentration is increased from 10^16^ to 10^18^ cm^−3^. It should be noted that the main reason for the dramatic drop in conductivity with doping concentration is that higher doping concentrations lead to increased porosity in SiNWs fabricated with MACE (Figure 3b,c,d). The dopant atom sites act as preferred locations for pore formation [19,23,26,27]. In comparison to the internal NW porosity, the phonon-impurity scattering at higher doping concentration has a much smaller impact on the thermal conductivity [2,12].

**Figure 4:**
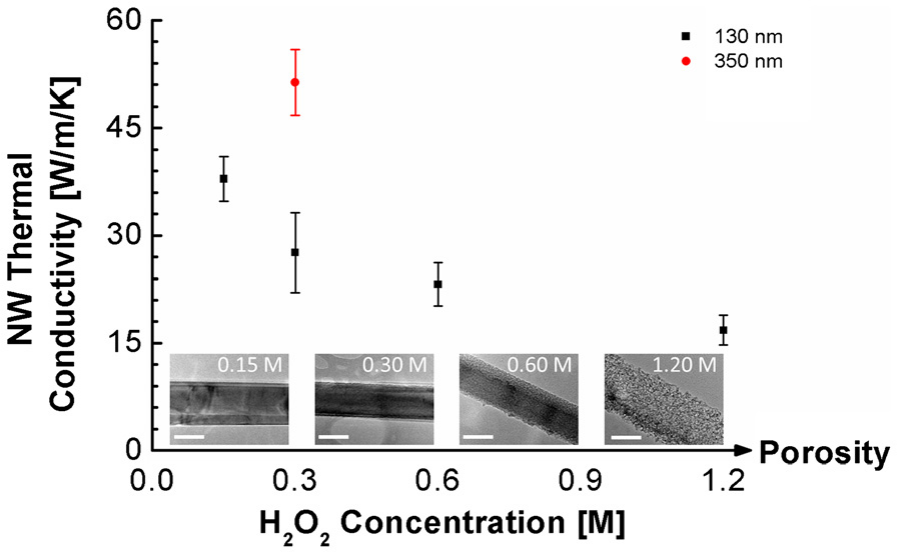
**Thermal conductivity of small-diameter (approximately 130 nm) SiNWs (10**^**14**^**cm**^**−3**^**) as a function of porosity.** For comparison, the thermal conductivity of the large-diameter SiNW etched at the same condition is shown as the red circle. Increasing nanowire porosity is realized by increasing the H_2_O_2_ concentration during MACE, as evidenced by the inset TEM images. The scale bars on all the TEM images are 100 nm.

The thermal conductivities of SiNWs with small diameters (*d*_avg_ ≈ 130 nm) also decrease with increasing porosity (Figure 4), similar to the large-diameter SiNWs. However, the thermal conductivity of these SiNWs is much smaller than that of large-diameter SiNWs of similar porosities (i.e., the same etchant solution, 0.3 M H_2_O_2_). Specifically, the thermal conductivity is reduced from 51 W/m/K for the large-diameter (*d*_avg_ ≈ 350 nm) SiNWs to 28 W/m/K for the smaller-diameter SiNWs (*d*_avg_*≈* 130 nm). This highlights the significant impact of phonon-external boundary scattering on the thermal conductivity at length scales that are smaller than the phonon mean free path. The additional reduction in thermal conductivity (to 17 W/m/K) with increasing H_2_O_2_ concentration for the smaller-diameter SiNWs indicates that the increasing internal porosity also has a significant impact on the thermal conductivity.

## Conclusions

In summary, we measured the thermal conductivity of SiNW arrays with various nanowire diameters, doping concentrations, surface roughness and internal porosities using a nanosecond transient thermoreflectance method. When the SiNW diameter (*d*_avg_ ≈ 350 nm) is larger than the phonon mean free path in the bulk silicon, the thermal conductivity shows little dependence on the doping concentration and surface roughness but decreases significantly with increasing porosity due to phonon scattering at the pore interfaces. In contrast, when the SiNW diameter (*d*_avg_ ≈ 130 nm) is smaller than the phonon mean free path, the thermal conductivity strongly depends on both the external boundary-phonon scattering and the internal pore interface-phonon scattering, leading to a significant reduction in the thermal conductivity for small-diameter SiNWs.

## Competing interests

The authors declare that they have no competing interests.

## Authors' contributions

JMW, AMM, KEG, and XLZ designed and interpreted the experiments. JMW and DRK fabricated the samples. JMW and PMR performed SEM and TEM characterization. AMM and MAP designed and carried out the thermoreflectance setup and measurements. All authors contributed to and approved the final manuscript.

## Supplementary Material

Additional file 1 **Error analysis of the thermal conductivity of vertical SiNW arrays.** An XLSX file showing detailed error analysis data for all the data reported. Click here for file
